# Participant Recruitment and Engagement in Automated eHealth Trial Registration: Challenges and Opportunities for Recruiting Women Who Experience Violence

**DOI:** 10.2196/jmir.6515

**Published:** 2016-10-25

**Authors:** Jane Koziol-McLain, Christine McLean, Maheswaran Rohan, Rose Sisk, Terry Dobbs, Shyamala Nada-Raja, Denise Wilson, Alain C Vandal

**Affiliations:** ^1^ Centre for Interdisciplinary Trauma Research Faculty of Health and Environmental Sciences Auckland University of Technology Auckland New Zealand; ^2^ Centre for Interdisciplinary Trauma Research Auckland University of Technology Auckland New Zealand; ^3^ Department of Biostatistics and Epidemiology Auckland University of Technology Auckland New Zealand; ^4^ Department of Preventive and Social Medicine University of Otago Dunedin New Zealand; ^5^ Taupua Waiora Centre for Māori Health Research Auckland University of Technology Auckland New Zealand

**Keywords:** eHealth, recruitment, dropout, intimate partner violence, Web-based trials

## Abstract

**Background:**

Automated eHealth Web-based research trials offer people an accessible, confidential opportunity to engage in research that matters to them. eHealth trials may be particularly useful for sensitive issues when seeking health care may be accompanied by shame and mistrust. Yet little is known about people’s early engagement with eHealth trials, from recruitment to preintervention autoregistration processes. A recent randomized controlled trial that tested the effectiveness of an eHealth safety decision aid for New Zealand women in the general population who experienced intimate partner violence (isafe) provided the opportunity to examine recruitment and preintervention participant engagement with a fully automated Web-based registration process. The trial aimed to recruit 340 women within 24 months.

**Objective:**

The objective of our study was to examine participant preintervention engagement and recruitment efficiency for the isafe trial, and to analyze dropout through the registration pathway, from recruitment to eligibility screening and consent, to completion of baseline measures.

**Methods:**

In this case study, data collection sources included the trial recruitment log, Google Analytics reports, registration and program metadata, and costs. Analysis included a qualitative narrative of the recruitment experience and descriptive statistics of preintervention participant engagement and dropout rates. A Koyck model investigated the relationship between Web-based online marketing website advertisements (ads) and participant accrual.

**Results:**

The isafe trial was launched on September 17, 2012. Placement of ads in an online classified advertising platform increased the average number of recruited participants per month from 2 to 25. Over the 23-month recruitment period, the registration website recorded 4176 unique visitors. Among 1003 women meeting eligibility criteria, 51.55% (517) consented to participate; among the 501 women who enrolled (consented, validated, and randomized), 412 (82.2%) were accrued (completed baseline assessments). The majority (n=52, 58%) of the 89 women who dropped out between enrollment and accrual never logged in to the allocated isafe website. Of every 4 accrued women, 3 (314/412, 76.2%) identified the classified ad as their referral source, followed by friends and family (52/412, 12.6%). Women recruited through a friend or relative were more likely to self-identify as indigenous Māori and live in the highest-deprivation areas. Ads increased the accrual rate by a factor of 74 (95% CI 49–112).

**Conclusions:**

Print advertisements, website links, and networking were costly and inefficient methods for recruiting participants to a Web-based eHealth trial. Researchers are advised to limit their recruitment efforts to Web-based online marketplace and classified advertising platforms, as in the isafe case, or to social media. Online classified advertising in “Jobs–Other–volunteers” successfully recruited a diverse sample of women experiencing intimate partner violence. Preintervention recruitment data provide critical information to inform future research and critical analysis of Web-based eHealth trials.

**ClinicalTrial:**

Australian New Zealand Clinical Trials Registry (ANZCTR): ACTRN12612000708853; https://www.anzctr.org.au/Trial/Registration/TrialReview.aspx?ACTRN=12612000708853 (Archived by WebCite at http://www.webcitation/6lMGuVXdK)

## Introduction

Violence against women is a global epidemic. It is estimated that one in every three women experiences physical or sexual violence by an intimate partner [1]. The negative impact of intimate partner violence (IPV) on the health and well-being of women and their children has been documented for over two decades [1,2]. While primary prevention efforts are required to reduce the prevalence of this human rights violation [3,4], it is also necessary to provide evidence-based essential services that promote the safety and well-being of those experiencing abuse [5]. However, women’s ability to access services is often constrained by the ongoing pattern of coercive and controlling behaviors that isolate a woman from both informal and formal support systems [6,7].

Recognizing the growing number of people who turn to the Internet to seek help [8,9], eHealth interventions have the potential to provide an accessible, safe, cost effective resource for women experiencing violence. eHealth interventions can provide a pathway for women to access health, justice, and civil society essential services. Innovative, interactive eHealth interventions for women who experience abuse are being developed and tested internationally [10-14]. In developing this evidence base, it is critical to examine the processes of recruiting women who are experiencing abuse for Web-based trials. In this study we examined the recruitment experience for the New Zealand isafe trial [15] that tested a Web-based safety decision aid for women who experience abuse.

### Help Seeking Among Women Who Experience Abuse

Qualitative research findings document that women implement a range of both active and passive strategies to keep themselves and their children safe from a partner’s coercive controlling behaviors [16-18]. They often tell friends, family, or coworkers (informal networks) about the abuse they are experiencing. These informal sources of support, however, often lack the understanding and skill to provide helpful responses [7,19]. Compared with disclosing abuse within informal networks, disclosing abuse within a formal service is less common. In countries with high rates of violence against women, and attitudes that condone abuse, less than 2% of women may access formal services [20,21]. In contrast, a 2003 survey of women in two New Zealand regions found that 52% reported seeking help from one or more formal services, although 40% of abused women reported that no one had tried to help them [22]. Barriers to seeking formal assistance reported by women include, for example, fear of repercussions from their partner, lack of trust, lack of confidentiality, fear of their children being removed, fear of deportation, potential loss of financial security (eg, if their partner is jailed), self-blame, feeling stigmatized, and the desire to not bring shame to themselves and their family [19,23-27]. Some women may have disclosed abuse in the past but received a response that minimized their abuse, was judgmental (victim blaming), or gave simplistic advice to leave their partner [28]. Barriers to effective help seeking are compounded when women experience multiple inequities. Such historical, social, cultural, structural, economic, and political contexts can result in mistrust of health institutions and racist and discriminatory responses that further entrap women [29,30]. Many of the root causes of violence directed against indigenous women, such as colonization resulting in historical trauma and racism, also contribute to and sustain unresponsive and potentially harmful institutional responses. This is most certainly the case for New Zealand Māori [31,32]. Web-based interventions have the potential to provide these women a confidential, culturally appropriate, nonjudgmental resource.

### Violence Against Women Intervention Research Recruitment

Recruiting women who experience violence for research testing intervention effectiveness can be particularly challenging for ethical, safety, and scientific rigor reasons [33,34]. Women are often recruited through government services or community agencies, which requires attention to relationship building with the agency, ideally with some provision for reciprocity [33]. Recruitment is generally advised by experts in the field to be done personally, in face-to-face, one-to-one encounters by trained, sensitive researchers who are skilled in managing unanticipated situations, and are ideally of the same culture as the women [33]. How this face-to-face recruitment process can be translated to the computer user interface in eHealth interventions has received little attention [35].

In a recent systematic review of studies testing advocacy interventions for women who experience violence [36], 8 of the 13 included studies recruited women from health care settings, 4 from domestic violence shelters (1 also recruited from social service agencies), and 1 from an urban community center. Of note, 2 Web-based trials were excluded from the systematic review due to not meeting the criteria for being advocacy based. Across the studies, reviewers identified variations in baseline type and severity of abuse, commitment to the relationship, participant age, ethnicity, and socioeconomic status, all of which can influence recruitment and dropout (attrition) and, in turn, study validity.

### eHealth Trial Recruitment Best Practice

Alongside the rapid growth of eHealth innovations are calls that programs be sufficiently tested. Dissemination models [37,38] and research standards [39] provide important resources for researchers, funders, and policy makers. Appreciating that “[randomized controlled trials] of Web-based interventions pose very specific issues and challenges,” the Consolidated Standards of Reporting Trials (CONSORT) -EHEALTH checklist was developed [39]. However, one aspect of eHealth trials that has garnered insufficient attention is the Web-based recruitment process. To understand Web-based recruitment and where there may be risk of bias, preintervention information is needed about the number of people who are potential participants, those who are eligible, those who consent, and those who complete baseline measures.

There is generally a lack of guidance regarding how external validity can be assessed and promoted in trials that involve Web-based recruitment. Scrutiny of recruitment and preintervention dropout experience in eHealth trials will inform our understanding of eHealth effectiveness and utility, trial representativeness, and the risk of fraudulent participation [40]. Few studies have addressed the representativeness of Web-based recruitment. In a review of 16 studies reporting dropout in Web-based interventions for psychological disorders, preintervention dropout (reported in 7 of the 16 studies) ranged from 4% to 52% [41]. Additionally, there is minimal theory to explain how potential participants engage with Web-based recruitment and interventions [35]. Liese and Beck [42] identified a pathway of individual and contextual factors that activate negative beliefs about the success of an intervention that are then hypothesized to lead to dropout. This does not take into account, however, participants’ expectations of an intervention effect, nor the altruistic aim of women who experience abuse wanting to help other women [41,43].

### isafe Trial

In this study we examined recruitment method effectiveness and early (preintervention) participant engagement for the isafe trial. The isafe trial was part of an international collaborative concurrent replication of the Internet Resource for Intervention and Safety (IRIS) study [11] that was modified for the Aotearoa New Zealand context [14]. The New Zealand trial, tailored for the New Zealand context [14], advanced the IRIS study by offering women fully automated Web-based trial recruitment, eligibility screening, and consent [44]. Both the IRIS and isafe studies had automated Web-based delivery of intervention, violence, and mental health assessments and retention procedures. While procedures to maximize isafe participant safety were paramount, and guided by explicit ethical principles, in this study we focused on recruitment and early participant engagement data. This information provides transparency of our experience for others to learn from, contributes to further refinement of eHealth study reporting guidelines, and informs critique of the isafe trial.

## Methods

We report and analyze isafe recruitment and early participant engagement as a case study [45], and include a qualitative narrative of the recruitment experience, as well as analysis of quantitative recruitment and engagement data. Specific aims included describing the recruitment experience, meeting recruitment targets, identifying preintervention engagement with Web-based registration and dropout rates, and examining the effects of study recruitment advertisements on an online community marketplace and classified advertising platform and their sustainability over time.

In the Web-based isafe trial, women were randomly assigned to a safety decision aid intervention or usual safety planning control website. Intervention components included (1) safety priority setting, (2) danger assessment [46], and (3) an individually tailored safety action plan. The control website included standardized (nontailored) safety planning and resource information. Self-reported primary outcome measures, depression and violence exposure, were collected at baseline and 3, 6, and 12 months after baseline. Women were provided a NZ $30 gift voucher at each measurement point in appreciation of their contribution. While the study protocol was previously published [15], in this paper we iterate and expand on the recruitment plan and automated registration process. The study protocol was approved by the Auckland University of Technology ethics committee (AUTEC 12/51) with trial registration (ACTRN12612000708853).

### A Priori Recruitment Plan

The target population for the isafe trial was women 16 years of age or older, residing in New Zealand, were English speaking, and were experiencing IPV in their current relationship. The target sample size was 340 women; we sought to enroll an average of 43 women per quarter, achieving the desired sample size over 24 months.

Recruitment was informed by several of our team principles. First, our principle to “be sensitive and inclusive of diversity” meant the program needed to reflect the diversity of women in Aotearoa New Zealand. The design and language used in isafe materials was influenced by women who participated in focus groups convened during the trial planning stage [14]. We also learned that, despite our being advised to collect numerous contacts to maximize retention in longitudinal studies [33], women in our focus groups resented being asked to identify more than one contact. Acknowledging inequities in Internet access, we also collaborated with Aotearoa People’s Network, a library collective that facilitates free Internet access across diverse New Zealand settings.

Second, our recruitment was guided by our principle to “maintain the cultural integrity of Māori within the *matatini* (diversity) of *iwi* /tribal differences.” The research team *kaumātua* (respected and recognized elder) and Māori team members (DW, TD) informed the development of recruitment plans and details such as ads and language. They also consulted *kanohi ki te kanohi* (face-to-face) with a range of Māori women, *iwi*, and service organization networks and attended conferences (eg, E Tu Whānau) to disseminate information about the isafe study.

Third, our principle to “act in a collaborative manner with our community and research partners” led us to consult with a range of advisors from local, regional, and national IPV service agencies (eg, SHINE, Women’s Refuge) and departments (Ministry of Social Development, It’s Not OK campaign). Team members provided presentations to agency staff and encouraged them to review the Web-based isafe tool using a guest password. Team members often had prior relationships with individuals within these agencies, who were supportive of the isafe resource and testing, and were willing to assist in recruitment.

The a priori recruitment plan was to initiate a dynamic stepped rollout of recruitment strategies over time, guided by recruitment data. The first phase of recruitment relied on the recent recruitment experience of the New Zealand Recovery via Internet from Depression (RID) trial. In the RID trial, short advertisements included in health education television programming (Health TV; Healthy Life Media Ltd, Auckland, New Zealand) in medical waiting rooms proved an effective recruitment method. For isafe, we developed two short video ads to run on televisions in the waiting rooms of 53 primary health care practices, 4 accident and emergency centers, and 3 emergency departments across New Zealand that subscribed to Health TV. Approximately half (28) of the practices served a predominantly Māori population. One or the other of the 30-second ads was to run once every 20 minutes for a period of 4 weeks. Information about the study was included in Health TV newsletters, and the research team communicated with each site offering flyers and isafe referral cards for display in their waiting rooms to supplement the television ad. The primary method of recruitment for the IRIS study was community ads on craigslist [11], which is not commonly used in New Zealand. While the team considered recruiting through social media sites such as Facebook, we were reluctant to do so due to security and confidentiality concerns. In addition to Health TV, the first phase of recruitment included plans for distribution of digital, print, and face-to-face recruitment ads. We prepared both mainstream and Māori-focused ads.

We monitored recruitment weekly, including review of Google Analytics (Google, Mountain View, CA, USA) reports and the isafe registration website. Summary data were available every 6 months in open Data Monitoring Committee reports. Recruitment methods were documented in a log and costs recorded. We budgeted NZ $17,000 for recruitment costs. This included $10,000 for production of Health TV ads and $4500 for running the ads. An additional $2500 was budgeted for other print and media recruitment methods.

### Web-Based Automated Registration Pathway

The automated registration pathway included the following 6 steps.

#### Welcome Page

People who found their way to the isafe website [44] were presented a welcome screen that provided a simple “Kia ora and welcome to the isafe study” message followed by 3 questions: “Are you a woman who is worried about your relationship?”, “Are you afraid of your partner sometimes?”, and “Do you sometimes wonder if you are safe?” From the welcome page, interested women were directed to click Sign Up to learn more about the study.

#### Sign Up Page

The sign up page provided study participant information that included, for example, the invitation to participate, purpose of the study and what would be involved, risk and benefits, confidentiality information, and how to contact the research team if site visitors had any questions. At the end of the participant information was the text “Thank you for carefully reading this information. If you are interested in taking part in this study press Next.” Clicking Next took them to the eligibility assessment.

#### Eligibility Page

On the eligibility page was the text “Please check the following to ensure that you are eligible to participate. This study was developed for women who are experiencing abuse in their current relationship. Please tick all that apply.” Items included the following: (*a*) In the last 6 months, I have been hit, kicked, punched, choked, or otherwise physically hurt by my current partner, (*b*) In the last 6 months, my partner has forced me into sexual activities or coerced me into sexual activities with threats, (*c*) In the last 6 months, my partner has threatened to harm me physically, (*d*) In the last 6 months, I have felt unsafe in my relationship, (*e*) I am a female, (*f*) I am 16 years or older, (*g*) I have computer access that is safe, (*h*) I have Internet access that is safe, and (*i*) I have an email address that is safe. Women who selected 1 or more of the abuse items (*a* – *d*) and each of the remaining items (*e–i*) advanced to the consent page. Those who did not meet eligibility criteria were thanked and referred to the resource page for general information; to reduce the risk of fraudulent entries, ineligible site visitors were blocked from using the browser Back button to return to the eligibility criteria. Women without a safe email were provided instructions on how they could obtain one.

#### Consent Page

Women who met the eligibility criteria completed the consent process by ticking their agreement to each of the consent items, ending with “I agree to be in this study.” Then, participant information was collected, including name, address, date of birth, safe email address, any special safety instructions for follow-up communications, and an alternative safe contact to facilitate retention. Women were also asked where they had learned about isafe (source of referral) and the number of children living in their home under the age 18 years that they were responsible for.

#### Automated Validation

We used a validation process to minimize the risk of fraudulent participant entry (such as duplicate entry). Validation was automated by matching the consenting participant’s name and address against the New Zealand Electoral Roll file (dated May 9, 2012) or by manual validation. Manual validation was completed by a research team member conducting a logic check of participant information (such as birthday) against information gathered by Google and Facebook searching or alternatively by sending an email request for confirmation of electoral roll status from the participant.

#### Automated Enrollment

Once validated, women were issued an automated email with their username, password, and the secure website address. For women validated by research staff, there was a delay from consent to email from an hour up to 2 days. Women had a 6-week window to enter the secure website and complete the baseline survey. Automated reminder emails were sent during the 6-week window. Once a woman completed the baseline measures she was considered to be accrued. This was the end of the preintervention phase of recruitment.

Team members received automated email notifications as potential participants progressed through the registration process. The trial registration database allowed team members to monitor participants’ progress from registration through to the 12-month follow-up.

### Data Collection and Analysis

Data sources included the study recruitment log, study financials, weekly Google Analytics reports, online marketing website visit reports, isafe registration administration data, and isafe metadata. Our analysis began with an examination of our study recruitment log to produce a narrative of the recruitment journey. We then examined people’s engagement in the isafe website, beginning with hits documented in Google Analytics (if country = New Zealand and acquisition = new user) and progressing through the preintervention pathway (using isafe registration administration data), noting dropouts. We next calculated the rates of accrual per month and day and compared accrual rates during the school holiday and non-school holiday periods. We report participant characteristics (age, children, referral source, and deprivation quintile based on consent address meshblock [47]) at both enrollment and accrual. The effectiveness of recruitment methods was considered, with cost per accrued participant calculated.

Given the novelty of recruiting through a series of location-specific Web-based online marketing website (Trade Me; Trade Me Ltd, Wellington, New Zealand) ads, we analyzed the efficiency of the ads by location and frequency of ads. The cumulative participant accrual versus number of ads for each region was graphed. We used the Koyck model [48,49], which links ads and sales in the econometric literature, to investigate the direct and lag effects of ads on accrual. The model was implemented using negative binomial regression with logarithmic link, accounting for regional variation with random effects. We investigated effects from the number of ad campaigns run in a region and school holidays. The analysis was undertaken with R version 3.2.0 (R Foundation for Statistical Computing), using the glmmADMB package [50].

## Results

### Recruitment Experience

The isafe recruitment experience can be easily separated into two distinct periods that we describe as *challenging* and *opportunities*.

#### The Challenging Period: September 17, 2012 to May 20, 2013

With the launch of the isafe trial on September 17, 2012, the a priori recruitment plan was implemented. The isafe ad ran in waiting room televisions for a 6-week period (September 17 to October 31, 2012). Study recruitment information was distributed nationally to IPV service agencies. Māori and mainstream recruitment leaflets and flyers were distributed through networks. Both IPV and general health agencies posted isafe links on their websites.

Our initial recruitment efforts were unsuccessful. With each passing month, we expanded our recruitment reach through additional digital, print, and face-to-face networks. We posted our video ad on YouTube (YouTube LLC, San Bruno, CA, USA) and increased the number of websites that provided a link to isafe (though many were time limited). We initiated additional newspaper advertisements. Flyers and ads in e-newsletters were distributed through universities and district health boards. Overall, these efforts were time and resource intensive—and ineffective.

At the end of 8 months, only 15 women had enrolled. The recruitment rate between September 17, 2012 and May 20, 2013 was approximately 2 women per month and made us question the feasibility of conducting the study. To develop an alternative recruitment strategy, we consulted with the university communications and marketing team, convened a recruitment think tank lunch to which we invited health promotion students, and consulted IRIS researchers (N Glass PhD, RN and A Clough, written and oral communication, April 2013) again; they iterated that they attributed their recruitment success to accessing women directly through an online classified advertisements website (craigslist; Craigslist Inc, San Francisco, CA, USA).

#### The Opportunities Period: May 21, 2013 to August 31, 2014

The team recognized the need to directly reach a wider audience of women. The leading online marketplace and classified advertising platform in New Zealand is Trade Me. While initial investigations had not identified a suitable section on the site for a research notice, we decided to trial an ad for “Research Study on Safety in Relationships” in the “Jobs–Other–volunteers” section. The ad included the mention “You will be reimbursed for your time.” Beginning May 21, 2013, a 4-week ad ran in five New Zealand locations. This short trial resulted in 21 participants: the opportunities phase had begun. While we expected that some might confuse the trial with a work opportunity, that was not the case. Review of our study log of phone calls (to our free phone study number) and emails identified only two queries about a work opportunity, both from men. Thereafter, at regular intervals, an additional 56 isafe ads were placed across 32 New Zealand cities, towns, and localities. We purposefully selected localities over time based on population size, proportion of Māori and Pacific people, high IPV rates (Recorded Crime Victim Statistics—Victimisations data generated from Statistics New Zealand [51]), and a large rural component.

During the opportunities phase, other recruitment efforts continued. For example, a collaboration between the isafe team and Auckland New Zealand Police resulted in a 3-month isafe recruitment drive (December to February 2014); New Zealand Police agreed to refer women to isafe during routine family violence callout follow-up visits. A second collaboration involved the Auckland Regional Community Alcohol and Drug Services, who agreed to post flyers and make isafe referrals to their clients.

**Figure 1:**
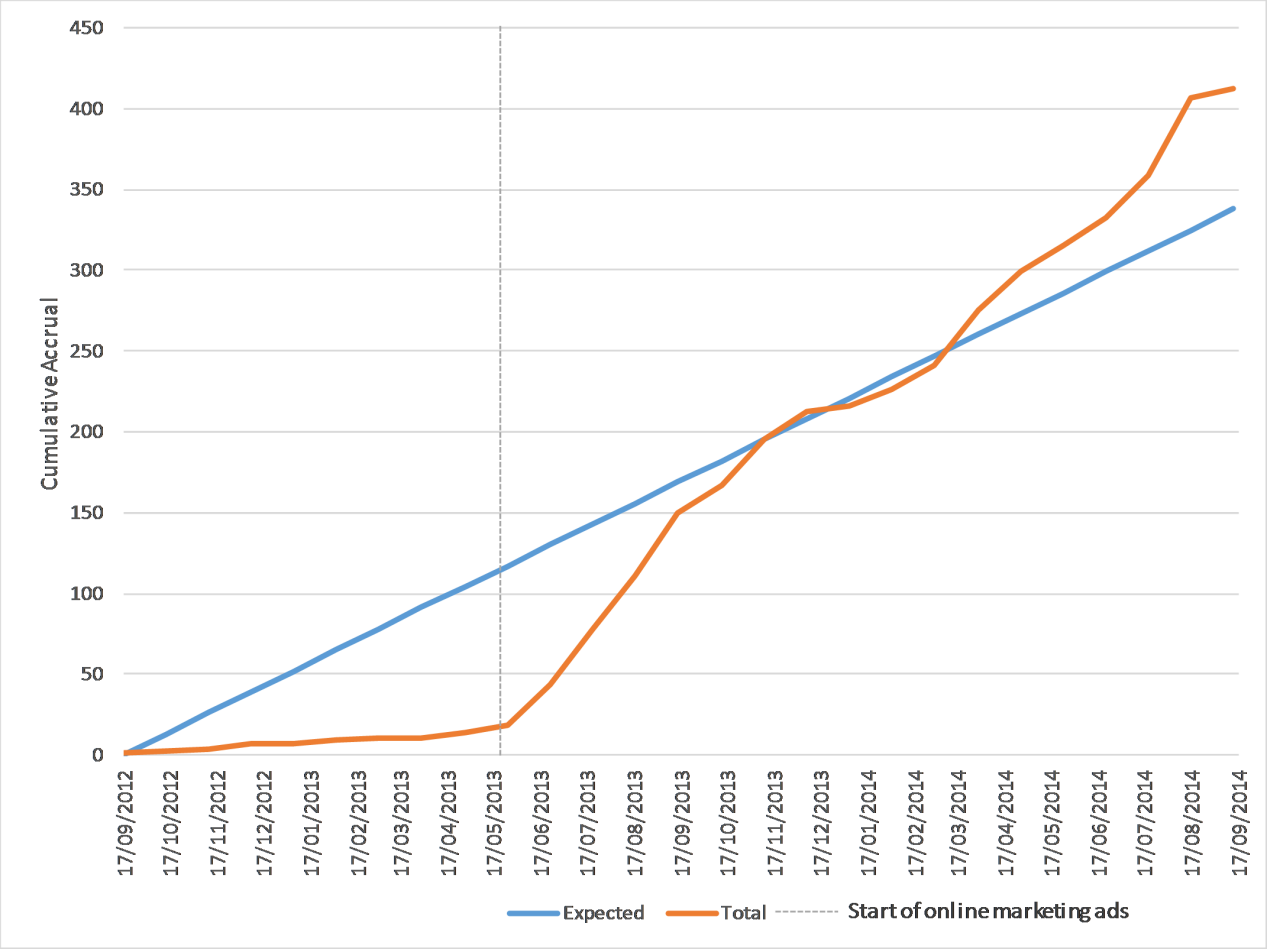
Expected and actual accrual to the isafe study, September 17, 2012 to August 31, 2014.

At the conclusion of the Trade Me ad campaign, by the end of August 2014, a total of 412 women had been accrued to the isafe study, exceeding our recruitment target of 340 well within the allocated timeline (Figure 1). The recruitment rate increased from 2 women per month during the challenging period to 25 women per month during the opportunities period (May 21, 2013 to August 31, 2014).

### Preintervention Participant Engagement

**Figure 2:**
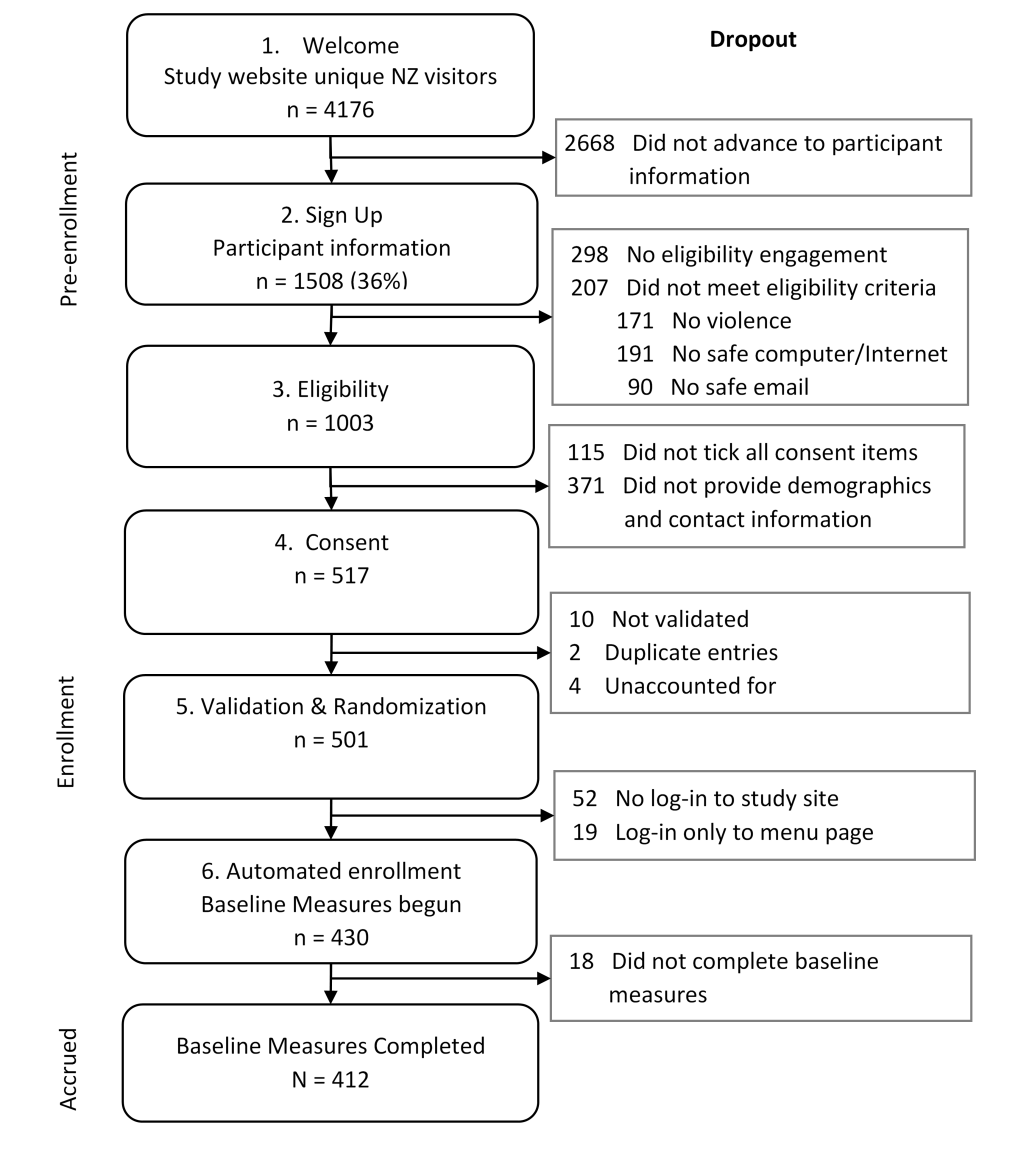
The isafe website preintervention participant engagement and recruitment.

Figure 2 outlines preintervention engagement based on the automated registration steps. The study website had over 4000 unique visitors, with 36.11% (1508/4176) reviewing the study participant information and 31.15% (1301/4176) engaging with the eligibility criteria. Among the 1003 women meeting eligibility criteria, 51.55% (517) consented to participate. Among the 501 women who enrolled (consented, validated, and randomized) in isafe, 412 (82.2%) were accrued (completed all baseline assessments). Among the 89 women who dropped out between enrollment and accrual, the majority (n=52, 58%) never logged in to the allocated isafe website. We acknowledge the nonstandard occurrence of baseline assessment after randomization, attributable to technical constraints.

Approximately 1 in every 10 unique visitors to our website became an accrued participant. The overall accrual rate per day was 0.58 (412 participants/713 days). During the 42 days of summer holiday (December 20 to January 10 over 2 years), no participants were recruited.

### Preintervention Participant Characteristics

**Figure 3:**
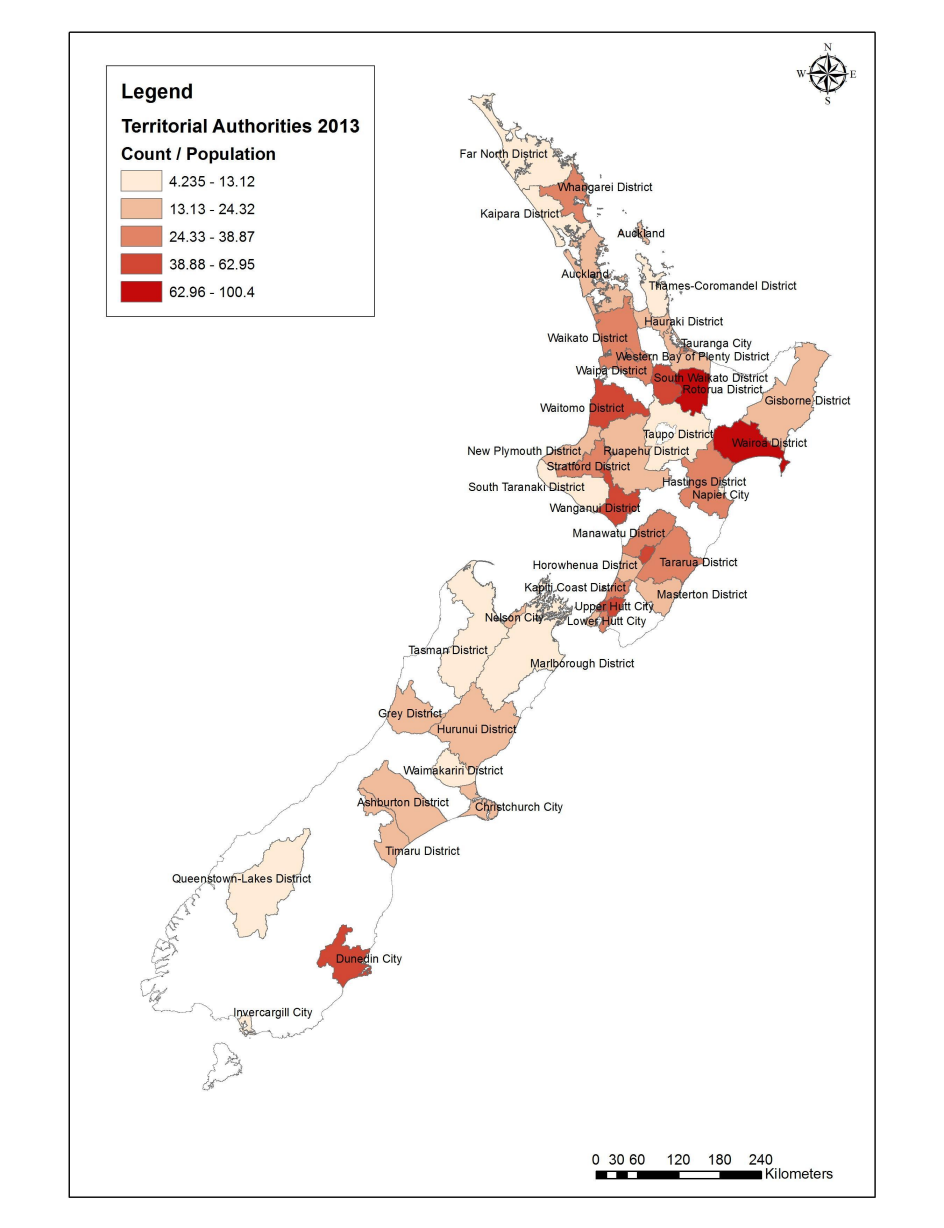
isafe study accrual rates across New Zealand territorial authorities.

Table 1 provides the characteristics of those who completed enrollment and those who went on to be accrued. Based on information provided at the time of consent, we noted no important differences between enrolled and accrued women in regard to age, having one or more children in the home, reporting two or more types of violence (among the four eligibility criteria), or deprivation. Accrued women were typically young, experienced multiple types of violence, and lived in higher-deprivation neighborhoods. Among accrued women, 27% self-identified as Māori (14.9% of people living in New Zealand identify with Māori ethnicity [52]). Women from most territorial regions in New Zealand were represented (Figure 3).

**Table 1:** Characteristics of enrolled versus accrued participants in isafe.

Characteristics		Enrolled (consented) (n=501)	Accrued (completed baseline measures) (N=412)
**Age in years**			
	Range	16–65	16–59
	Mean (SD)	31.2 (10.0)	30.8 (9.9)
One or more children in the home, n (%)		230 (46)	186 (45)
**Violence (among 4 types of violence), n (%)**			
	One type of violence	132 (26.3)	110 (26.7)
	Two or more types of violence	369 (73.7)	302 (73.3)
**Referral source, n (%)**			
	Online marketing ad (Trade Me)	377 (75.2)	314 (76.2)
	Friend or relative	59 (11.8)	52 (12.6)
	Domestic violence service provider	19 (3.8)	10 (2.4)
	Health TV/medical clinic	9 (1.8)	9 (2.2)
	CADS^a^	4 (0.8)	3 (0.7)
	Newspaper	3 (0.6)	3 (0.7)
	New Zealand Police	2 (0.4)	2 (0.5)
	YouTube	2 (0.4)	1 (0.2)
	Other	26 (5.2)	18 (4.4)
**Deprivation quintiles (based on consent address), n (%)**			
	1 (lowest deprivation)	44 (8.8)	42 (10.2)
	2	75 (15.0)	62 (15.0)
	3	105 (21.0)	81 (19.7)
	4	115 (23.0)	97 (23.5)
	5 (highest deprivation)	149 (29.7)	123 (29.9)
	Unknown	13 (2.6)	7 (1.7)
**Ethnicity (self-identified; could select >1 ethnicity), n (%)^b^**			
	Māori		113 (27.4)
	Pacific		42 (10.2)
	Asian		42 (10.2)
	New Zealand European		297 (72.1)

^a^CADS: Community Alcohol and Drug Services.

^b^Data not collected at time of enrollment.

**Table 2:** Recruitment efficiency for the isafe study.

Recruitment type	Staff resource	Direct cost (NZ $)	No. of accrued participants	Cost per participant (NZ $)
Health clinic television ads (Health TV; development and running)	Low	12,256	9	1362
Online marketing ads (Trade Me)	Low	6831	314	22
Print ads (newspapers)	Low	829	3	276
Flyers (printing)	High	910	Unknown	Unknown
Networking with service providers	High	230	10	23
Not assigned to a costed recruitment method			76	unknown
TOTAL		21,056	412	51

**Table 3:** Characteristics of accrued participants (N=412) in isafe by referral source.

Characteristics		Referral source		
		Friend/relative (n=52)	Trade Me (n=314)	Other (n=46)
**Age in years**				
	Mean (SD)	30.83 (9.06)	30.68 (10.11)	31.72 (9.8)
	Range	17–57	16–59	17–57
One or more children in the home, n (%)		19 (36.5)	148 (47.1)	19 (41.3)
Two or more types of violence, n (%)		38 (73.1)	230 (73.2)	34 (73.9)
**Deprivation quintile, n (%)**				
	1 (lowest deprivation)	2 (3.8)	34 (10.8)	6 (13.0)
	2	8 (15.4)	48 (15.3)	6 (13.0)
	3	6 (11.5)	68 (21.7)	7 (15.2)
	4	15 (28.8)	72 (22.9)	10 (21.7)
	5 (highest deprivation)	21 (40.4)	85 (27.1)	17 (37.0)
Ethnicity self-identified as Māori		21 (40.4)	83 (26.4)	9 (19.6)

### Recruitment Methods

A total of 3 of every 4 women (76%) recruited to isafe identified Trade Me as their referral source (Table 1). The next most common referral source was through friends and family (12.6%). We do not know how friends and family heard about isafe, whether from seeing an ad on Health TV or Trade Me, for example. Recruitment costs totaled NZ $21,056 ($4056 overspend). The cost per participant ranged from $1362 for the development and running of television ads in medical clinics, to $22 for online marketing ads (Table 2). The online marketing ads were both efficient in cost and staff time. While networking direct costs were low, the required staffing resource was significant.

There were some notable differences in participant characteristics by referral source (Table 3). Women recruited through a friend or relative were more likely to self-identify as Māori and live in the highest-deprivation areas. Women recruited through Trade Me were more likely to have children in the home and less likely to live in the highest-deprivation areas.

### Online Marketing Ad Effects

We examined the online marketing website (Trade Me) ad impact on accrual weekly during recruitment. Research staff attributed (matched) accrued women to a Trade Me ad based on location and date of accrual (research staff were blinded to women’s self-report of referral source). Research staff attributed a total of 327 (13 more than what women reported) accruals to Trade Me ads. We placed 61 ads in 32 locations throughout New Zealand during the period May 2013 to August 2014. Individual locations had between 1 and 5 ads. The ads resulted in a total of 65,067 views on the marketing website. Individual ads produced between 0 and 9 participants while the ad was running.

The lag effect duration of ads past the publication period was estimated at 0.3 day (95% CI –0.3 to 1.2), not significantly distinct from an absence of actual lag effect (*P*=.3). We therefore refitted the model without the lag effect, but included the number of ads previously run in the region and an indicator for school holidays, for neither of which we could account in the original Koyck model. The base recruitment rate was estimated by the model at 0.017 participant per 100,000 person-weeks (95% CI 0.011–0.024). On average, ads increased the accrual rate by a factor of 74 while they were running (95% CI 49–112). Running several ads over time in the same region was associated with a significant decrease of the accrual rate, by a factor of 0.71 after each ad campaign (95% CI 0.64–0.78). School holidays were found not to be significant (*P*=.21) and were not retained in the model. There was fairly strong regional variation, with fitted regional random rate ratios ranging from 0.17 to 6.2.

## Discussion

The recruitment target for the Web-based isafe intervention trial for women who experience abuse was to accrue 340 women over a 24-month period. This target was achieved. On the automated study registration website, within 23 months, 4176 people visited the website, 501 women enrolled, completing the consent and validation process, and 412 (82.2%) women were accrued, having completed baseline measures. However, these simple recruitment statistics mask the challenges to and opportunities for recruitment that were experienced in the preintervention recruitment phase of the study.

The recruitment rate during the 8-month *challenging* period (2 women per month) represented an inefficient, financially wasteful period of recruitment print ads, website links, and networking. In contrast, the recruitment rate during the 16-month *opportunities* period (25 women per month) was valuable in both efficiency and cost. Job ads for volunteers in the largest available online consumer marketing and classified advertising website in New Zealand (Trade Me) were viewed over 65,000 times; 3 of every 4 participants identified the job ad as their source of referral to isafe. Staff time in managing and monitoring the advertising process was minimal. Interestingly, we estimate that if we had had administrative capacity to monitor high flows of participants into the trial, by running a blitz of ads across New Zealand every 6 weeks, we might have achieved our sample size within 6 months. Our challenges and opportunities experience is similar to that reported by Loxton et al [53], where the average number of daily responses for recruiting young women to the Australian Longitudinal Study on Women’s Health increased 5-fold with the introduction of targeted Facebook advertisements. The current I-DECIDE Australian trial is also using Facebook and achieving expected recruitment rates [10].

When targeting participants within a large population, marketing websites or social media recruitment methods are advised. The decreasing effectiveness of ad campaigns as they are repeated, either through exhaustion of the recruitment population or lessened sensitivity to the ads, is a factor to bear in mind. Other recruitment considerations include holiday periods, the type of topic that is being researched, characteristics of the target group, and digital media tools that the group are most likely to use. Working with colleagues experienced in social and health marketing fields is also helpful. One of the strengths of Web-based studies is being able to recruit diverse populations. In our case, we successfully recruited women from across New Zealand; 1 in 4 women self-identified as Māori, and women living in high-deprivation neighborhoods were overrepresented. Of note, our preintervention data highlight the important contribution of friends and family in referring high-risk women to research, particularly for Māori women.

The 18% isafe preintervention dropout rate from enrollment (consent, validation, and randomization) to accrual (completion of baseline measures) is just below the weighted average of 21% (range 4% to 52%) reported by Melville et al [41] for trials of Web-based interventions for psychological disorders. Calculating preintervention dropout rate from earlier steps in the automated Web-based registration process, however, results in dropout rates of 59% (from eligibility assessment) to 20% (from consent criteria), indicating the importance of clarifying at which point dropout is being measured. Improved linkage between registration and intervention (and control) websites may reduce dropout between enrollment and accrual in future studies.

### Limitations

This case study shares the experience of one trial in New Zealand. The available social media and populist platforms vary internationally and are sure to change over time. For the isafe study, while networking with community agencies was less valuable for recruitment, it was valuable in promoting referral links from the isafe program to services. Clarity of purpose for partnering with agencies and organizations will contribute to the efficiency and safety of the trial and its participants, as well as improve the overall trial quality and knowledge transfer.

In this case study, we collected significant standardized information on preintervention engagement with our Web-based study registration site. We do not know, however, the characteristics of people who visited the site, but dropped out prior to providing contact information. Nor do we know the reasons why people may have visited the site, such as for curiosity or help seeking, but disengaged.

In addition, the comparison of enrolled and accrued participant characteristics by referral type was based on participants’ self-report. In contrast, our online marketplace (Trade Me) analysis of effect modelling was based on research staff attributing accrued participants to an ad during weekly recruitment reviews based on participant and ad location and timing. This process underestimated the number of participants recruited through Trade Me by 13. In addition, our assignment of deprivation is based on the address provided by the study participant. This likely includes error due to variation in socioeconomic status within neighborhoods, the housing instability of women who experience violence [54], and women electing to enter an address other than their own due to issues of privacy, safety, or mistrust.

### Conclusions

Populist website recruitment methods can successfully recruit a diverse sample of participants for studies addressing sensitive topics such as violence against women. We hope our transparency in reporting preintervention participant engagement will influence others to do the same during this period of rapid growth in the number of eHealth intervention trials with automated Web-based registration. As suggested by van Gemert-Pijnen et al, “Now it is time to recapitulate the lessons learnt. We need a holistic approach to e-health development that is evidence-based and people-centred, that takes into account how people live within their own environments and that focuses on responding to stakeholders’ needs and improving care” [55]. With sensitivity and research rigor, Web-based interventions have the potential to provide women a confidential, culturally appropriate, nonjudgmental resource to support their pursuit of safety and well-being.

## Abbreviations

CONSORT: Consolidated Standards of Reporting Trials
IPV: intimate partner violence
IRIS: Internet Resource for Intervention and Safety
RID: Recovery via Internet from Depression
